# Mutated genes on ctDNA detecting postoperative recurrence presented reduced neoantigens in primary tumors in colorectal cancer cases

**DOI:** 10.1038/s41598-023-28575-3

**Published:** 2023-01-24

**Authors:** Satoshi Nagayama, Yuta Kobayashi, Mitsuko Fukunaga, Shotaro Sakimura, Keishi Sugimachi, Shin Sasaki, Takaaki Masuda, Ken-ichi Mafune, Masanobu Oshima, Tatsuhiro Shibata, Yutaka Suzuki, Koshi Mimori

**Affiliations:** 1 Department of Gastroenterological Surgery, Cancer Institute Hospital, Japanese Foundation for Cancer Research, Gastroenterological Center, 3-8-31 Ariake, Koto-ku, Tokyo, 135-8550 Japan; 2 Departmnet of Surgery, Uji-Tokushukai Medical Center, Kyoto, 611-0041 Japan; 3 Department of Surgery, Kyushu University Beppu Hospital, 4546 Tsurumihara, Beppu, 874-0838 Japan; 4 Department of Surgery, Coloproctology Center Takano Hospital, 3-2 Ooe, Kumamoto, 862-0971 Japan; 5 Department of Hepato-Biliary and Pancreatic Surgery, Kyushu Cancer Center, 3-1-1, Notame, Minami-ku, Fukuoka, 811-1395 Japan; 6 Department of Coloproctological Surgery, Japanese Red Cross Medical Center, 4-1-22 Hiroo, Shibuya-ku, Tokyo, 150-8935 Japan; 7 Department of Surgery, Ofuna Chuo Hospital, 6-2-24 Ofuna, Kamakura, 247-0056 Japan; 8 Division of Genetics, Cancer Research Institute, Kanazawa University, Kadoma-cho, Kanazawa, 920-1164 Japan; 9 Laboratory of Molecular Medicine, Institute of Medical Science, Human Genome Center, University of Tokyo, 4-6-1, Sirokane-dai, Minato-ku, Tokyo, 108-8639 Japan; 10 Medical Genome Sciences, Graduate School of Frontier Sciences, University of Tokyo, 5-1-5 Kashiwanoha, Kashiwa, 227-8561 Japan

**Keywords:** Cancer microenvironment, Gastrointestinal cancer, Tumour biomarkers, Cancer, Oncology

## Abstract

The detection and sequencing of the mutated ctDNA is one of the irreplaceable clinical measures in the postoperative management of colorectal cancer (CRC) cases. However, we are curious to comprehend the essential traits of mutated genes comprising metastatic sites out of whole mutated genes in primary sites. In the current retrospective study, we conducted target resequencing of ctDNA using 47 plasma samples and established a cancer panel carrying the commonly mutated genes between primary and recurrent tumors. We found that mutated genes in ctDNA indicated immune-resistance traits with respect to the impaired ability to present neoantigens by loss of expression or binding affinity to HLA in the primary tumor. Compared with the estimated neoantigens from all mutated genes in primary tumors, the neoantigen peptides from commonly mutated genes on the panel showed abundant expression but no binding affinity to HLA. Therefore, ctDNA mutations can be frequently and postoperatively detected to identify recurrence; however, these mutated genes were derived from immune-tolerated clones owing to the loss of neoantigen presentation in primary CRC tumors.

## Introduction

In general, we use circulating tumor (ct)DNA as a liquid comprehensive genomic profile (CGP) assay, which is not inferior to CGP tissue analysis in gastrointestinal cancers^1–5^. In addition, we can trace mutations in ctDNA to monitor the minimum residual tumors for identification of recurrence at the subclinical level. However, in terms of liquid biopsy using ctDNA, we have to comprehend the characteristics of the detected mutation in ctDNA derived from epithelial cells in the primary tumor nests to form recurrence postoperatively.

Considering the essential characteristics of the mutated clones that were chronologically and sustainably detected from the primary site to the recurrent site continuously, we assumed two possibilities. First, the mutated genes in ctDNA may be detected abundantly in tumors with high mutation allele frequency (MAF) or clonally expanded mutated genes that cover the entire primary tumor. These highly mutated or clonally mutated genes may be derived from the dominant cancer cells to promote cancer progression in primary colorectal tumors^6^. We previously disclosed that driver mutated genes, such as canonical oncogenes and suppressor genes, dominated the entire primary tumor region as the neutral evolution manner in advanced CRC cases^7,8^. In addition, we previously reported a case of CRC in which mutated *KRAS* was detected in ctDNA from primary and metastatic tumors simultaneously^9^, indicating a continuously higher MAF during longitudinal radical treatment.

Another possibility is that the localized host tumor immune response in primary tumors may affect the sensitivity to detect ctDNA in the circulation system. The tumor immune response in cancer microenvironment is comprised of CD8^+^ cytotoxic T lymphocyte, FOXP3^+^ CD4^+^ T regulatory cells, dendritic cells, macrophages, and cytokines. The several former studies touched the association between detectability of mutated ctDNA fragments and the host immunity^10–13^, however, they could not reach at any definitive conclusions. In terms of the association between the host immunity and the detectability of mutated ctDNA, the current study focuses on the presentation ability of neoantigens derived from somatic mutations in ctDNA, which is determined by the following two factors: the binding affinity of the diverse estimated neoantigens of mutated genes to human leukocyte antigen (HLA) and expression of tumor-specific RNA transcribed from mutated alleles. Both factors were indispensable for presenting neoantigens derived from mutated genes in ctDNA among all mutated genes in the primary tumor.

This study conducted target sequencing of ctDNA from 47 points in the clinical course of six cases of CRC with postoperative recurrence (CRCR) using a customized cancer panel for target resequencing of commonly mutated genes between primary and recurrent sites^14^ (Table 1). We calculated the binding affinity to HLA (half maximal inhibitory concentration [IC50]) and tumor-specific RNA expression of mutated genes among all mutated genes in primary tumors by in silico analysis. In the current study, we disclose how tumor immune response can affect the detectability of mutated ctDNA in the primary tumor.

**Table 1:** Information of 10 cases of colorectal cancer with recurrence (CRCR).

ID	Sex	Age	Primary tumor						Metastattic tumor		Mutated genes on each panel
			Tumor location	Size	Tumor differentiation	UICC stage	ly	v	Recrrence site	Months after surgery	
CRCR1	M	68	Sigmoid colon	40 × 40	Well differentiated	Stage II (T4N0M0)	1	1	Liver*	4.1	29 genes
CRCR2*	F	63	Rectum (Rb)	15	Well to moderately differentiated	Stage I (T1N0M0)	0	0	Lung	8.7	28 genes
CRCR3	M	33	Rectum (Ra)	40 × 40	Well differentiated	Stage II (T3N0M0)	1	1	Lung	M1: 20	54 genes
									Lung	M2: 35.6	
CRCR4	M	41	Sigmoid colon	23 × 19	Moderately differentiated	Stage I (T2N0M0)	1	2	Liver	24.2	36 genes
CRCR5*	F	60	Rectum (Ra)	30 × 30	Well differentiated	Stage II (T3N0M0)	1	1	Lung	12.6	26 genes
CRCR6	M	56	Rectum (Rb)	32 × 28	Moderately differentiated	Stage I (T2N0M0)	2	1	Lung	10.4	24 genes
CRCR7	F	73	Ascending colon	25 × 25	Moderately differentiated	Stage I (T1N0M0)	1	0	Liver*	M1: 5.3	65 genes
									Liver	M2: 16.1	
									Liver	M3: 18.8	
CRCR8	M	76	Sigmoid colon	28 × 20	Moderately differentiated	Stage II (T4N0M0)	1	2	Liver	M1: 6.1	25 genes
									Liver	M2: 11.1	
CRCR9	F	55	Sigmoid colon	35 × 35	Moderately differentiated	Stage II (T3N0M0)	0	2	Liver	2.9	36 genes
CRCR10	F	78	Rectum (Ra)	20 × 20	Well differentiated	Stage I (T2N0M0)	1	1	Lung*	M1: 6.6	120 genes
									Lung*	M2: 29.7	
											443 genes

We excluded CRCR2, CRCR3, CRCR6 and CRCR10 from further analysis, because of inadequate amount of plasma samples.

*No RNA Sequence data in 2 primary and 4 metastatic tumors.

## Results

### Landscape of mutated ctDNA using target sequencing in six CRC cases

**Figure 1:**
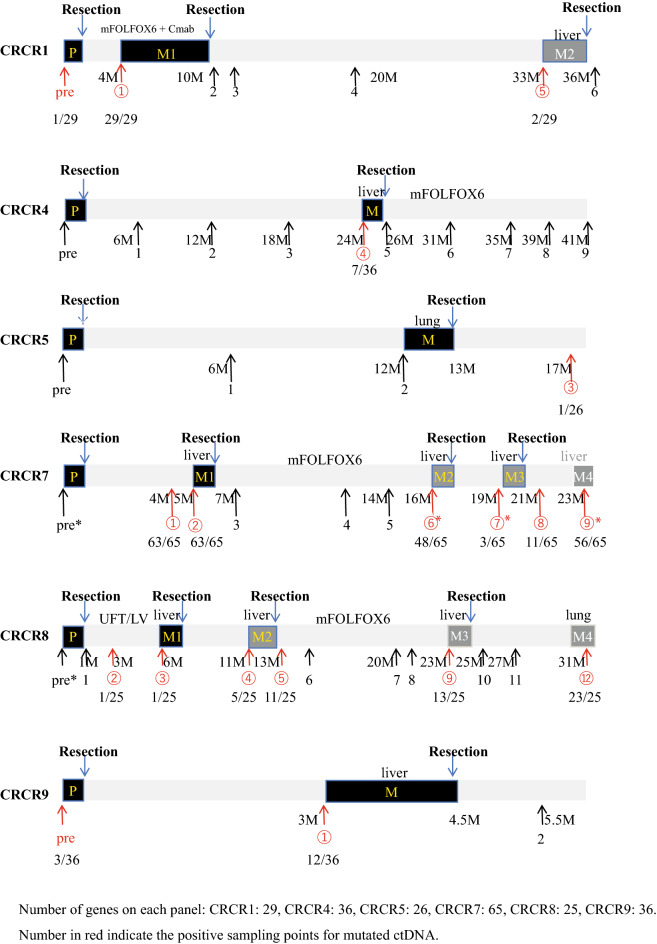
Plasma sampling from six cases of CRC with postoperative recurrence. Whole-exome sequencing and RNA sequencing of P (primary tumor) and M (metastatic tumor) were conducted. The number of mutated genes on each panel: CRCR1, 29; CRCR4, 36; CRCR5, 26; CRCR7, 65; CRCR8, 25; and CRCR9, 36. Numbers in the red circle indicate positivity for mutated genes in the ctDNA. We included the ratio of mutated genes to all genes in each cancer panel. Pre, preoperative plasma sample.

We applied ten primary tumors and ten postoperative recurrence sites to extract genomic DNA for whole-exome sequencing (WES) analysis, which was reported in our previous study^14^. We selected 443 commonly mutated genes between 10 primary sites and 10 recurrent (metastatic) sites to establish a cancer panel for target resequencing (Fig. S1). In addition, we added 35 significant canonical mutated genes. Out of those 35 genes, twenty-seven genes were overlapped with the 443 mutated genes from the current 10 cases. Therefore, as a consequence, 451 mutated genes were on the panel (Fig. S2). Unfortunately, we could not collect an adequate amount of plasma from four cases shaded areas in Table 1, such as CRCR2, CRCR3, CRCR6, and CRCR10, therefore, we excluded them from the target sequence analysis as the liquid biopsy. The clinical courses of the six cases, CRCR1, CRCR4, CRCR5, CRCR7, CRCR8, and CRCR9 involving 47 samples from primary or metastatic tumors are presented in Fig. 1. For example, in CRCR7, we detected 63 mutated genes out of 65 mutations on the panel (average AF of 63 genes: 0.146) at 4 M (➀) and 63 of 65 mutations (average AF of 63 genes: 0.172) at the diagnosis of metastasis (➁) (Fig. 1).

### Verification of ctDNA to capture commonly mutated genes between primary and recurrent tumors

**Figure 2:**
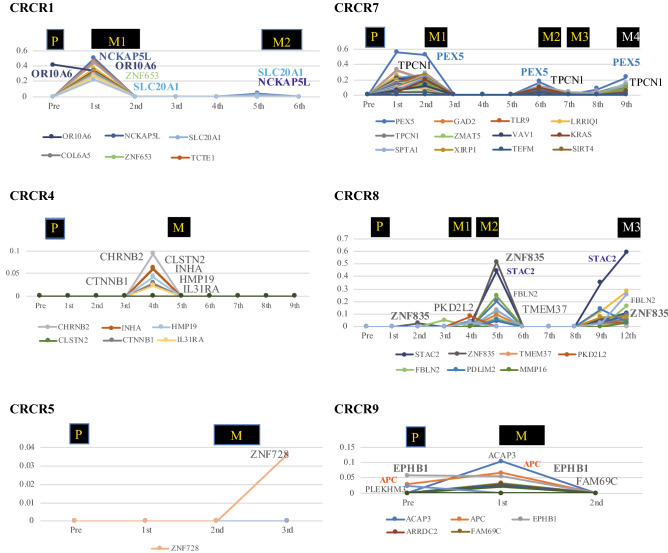
Alteration of mutation allele frequency of target genes. In CRCR1, *NCKA5L* and *SLC20A* showed mutations from M1 to M2, respectively. An *OR10A6* mutation was detected preoperatively and at M1. In CRCR4, *CTNNB1*, *CHRNB2*, and *CLST2* were frequently mutated in the M1 sample. In CRCR7, the MAFs of *PEX5* and *TPCN1* were detected multiple times with recurrent tumors. In CRCR8, *STAC2*, *ZNF835*, and *FBLN2* were altered along with M2 and M4. In CRCR9, a higher MAF of *EPHB1* was detected in preoperative and M1 samples.

In this retrospective study, it was essential to verify the accuracy of the current assay for implementing the target sequence of plasma ctDNA. We found that this assay system could capture mutated ctDNA genes using a cancer panel that carrying the commonly mutated genes between primary and recurrence sites. As shown in Fig. 2, CRCR1, CRCR4, CRCR7, CRCR8, and CRCR9 have candidate target genes with mutations that were detected repeatedly in ctDNA for tumor tracing throughout the postoperative clinical course. In CRCR7, the PEX5 gene^15^ was clearly captured multiple times by commonly mutated genes in primary and recurrent tumors.

### Comparison of neoantigen presentation ability between mutated genes in ctDNA and all mutated genes in primary tumors

We focused on the ability to present neoantigens derived from commonly mutated genes between primary and recurrent sites compared with whole mutated genes in primary tumors. Presenting neoantigens to activate the tumor immune response requires simultaneous estimation of the binding affinity of the mutated allele to HLA and the expression of the cancer-specific mutated allele. Major histocompatibility complex (MHC) restriction was examined by predicting the binding affinity of single nucleotide variants (SNVs) to HLA (using the analytical pipeline NetMHCpan)^16,17^ (Fig. S3). We extracted an altered read from the tumor RNA BAM file and measured the expression of tumor-specific mutated genes among all mutated genes in the primary sites as the scheme.

**Figure 3:**
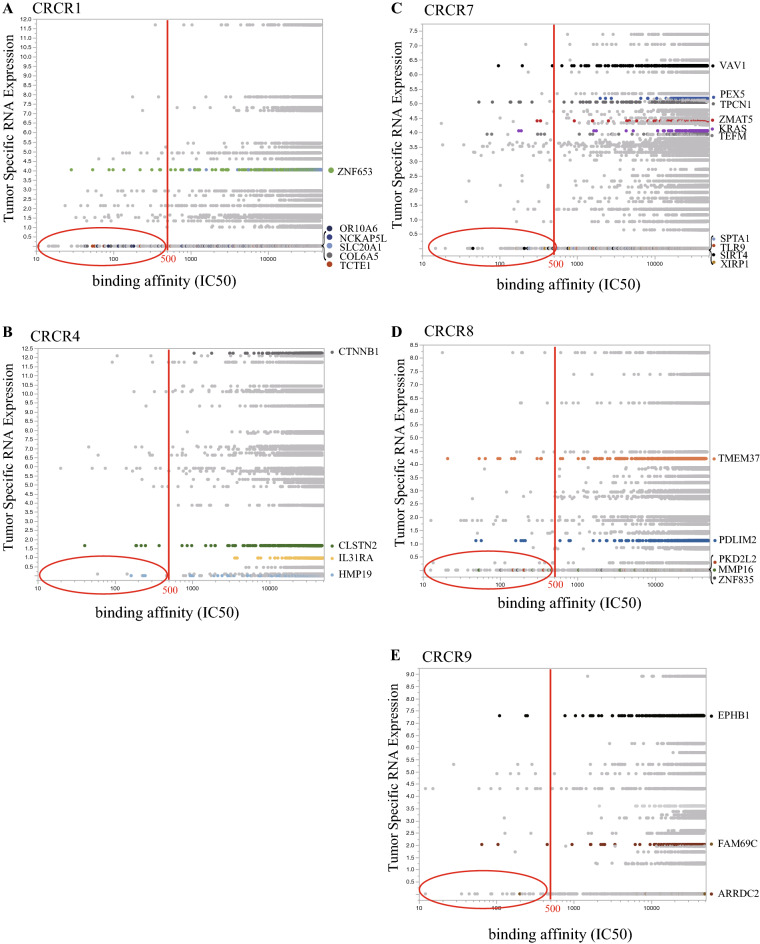
Immunogenicity of estimated NAG peptide in each mutated ctDNA gene. In terms of neoantigen analysis for MHC restriction of ctDNA, the binding affinity of peptide fragments to HLA-A, -B, -C was estimated using SNVs with WES data of primary sites (neBindingtMHCpan) **(X-axis)**. The binding affinity of peptides was calculated as the IC50. The estimated NAG peptide derived from an SNV within 500 nM (red line) of IC50 indicated weak binding affinity to HLA. In addition, tumor-specific RNA expression was extracted from the tumor RNA BAM file and evaluated **(Y-axis)**. Neoantigens with a high binding affinity but no expression were spotted in a red elliptic circle.

In CRCR7P, we found that tumor-specific mutated PEX5 RNA expression was significantly higher than the expression of all genes in the primary site (Table 2); however, there were no peptide PEX5 fragments within the high range of binding affinity (IC50 < 50 nM) among the estimated 6740 peptide fragments from 104 mutated genes. Therefore, the altered PEX5 must not be presented as a neoantigen peptide. In CRCR1P, mutated OR10A6 showed a higher binding affinity (20 [5.29%] of 378 peptides) to HLA than that of other mutated genes (p < 0.0001). On the other hand, the mutated OR10A6 gene was not presented as a neoantigen (Table 2); therefore, OR10A6^18^must not be presented as a neoantigen peptide. As shown in Table 2, representative mutated genes that could be chronologically traced by ctDNA showed either low binding affinities with HLA or low expression of mutated genes in a mutually exclusive manner. We plotted ctDNA mutated genes to demonstrate the minimized binding affinity to HLA and low expression of mutated transcripts in ctDNA (Fig. 3). Therefore, we assumed that chronologic ctDNA-detected mutated genes were derived from immune-tolerant cancer cells rather than cytolytic activity-inducing collapsed cancer cells.

**Table 2:** **C**omparison of binding affinity to HLA and expression of tumor specific RNA between representative detetected mutated genes in ctDNA and primary specific mutations.

	Mutated genes to estimate NAG	Low affinity	High affinity	Fisher	Tumor specific RNA expression	No-expressed	Expressed	Fisher
CRCR1P	OR10A6 in primary	358 (94.71)	20 (5.29)	< 0.0001*	Mutated OR10A6	378	0	
	All mutated genes in primary	23506 (99.02)	229 (0.98)		All mutated genes in primary	15498	7879	< 0.0001
	SLC20A1 in primary	378 (100)	0		Mutated SLC20A1	13 (3.44)	365 (96.56)	< 0.0001*
	All mutated genes in primary	23128 (98.93)	249 (1.07)	p = 0.0368	All mutated genes in primary	15863 (67.86)	7514 (32.14)	
CRCR4P	CTNNB1 in primary	378 (100)	0	ns	Mutated CTNNB1	0	378 (100)	< 0.0001*
	All mutated genes in primary	11312 (99.59)	46 (0.41)		All mutated genes in primary	4536( 39.94)	6822 (60.06)	
	CLSTN2 in primary	372(98.41)	6 (1.59)	p = 0.0034*	Mutated CLSTN2	376 (99.47)	2 (0.53)	
	All mutated genes in primary	11318 (99.65)	40 (0.35)		All mutated genes in primary	4160 (36.63)	7198 (63.37)	< 0.0001
CRCR7P	PEX5 in primary	378 (100)	0	ns	Mutated PEX5	10 (2.65)	368 (97.35)	< 0.0001*
	All mutated genes in primary	41754 (99.57)	180 (0.43)		All mutated genes in primary	20870 (49.77)	21064 (50.23)	
	TPCN1 in primary	371 (98.15)	7 (1.85)	p = 0.0012*	Mutated TPCN1	6 (1.59)	372 (98.41)	< 0.0001*
	All mutated genes in primary	41761 (99.59)	173 (0.41)		All mutated genes in primary	20874 (49.78)	21060 (50.22)	
CRCR8P	TMEM37 in primary	367 (97.09)	11 (2.91)	< 0.0001*	Mutated TMEM37	2 (0.53)	376 (99.47)	< 0.0001*
	All mutated genes in primary	22919 (99.40)	139 (0.60)		All mutated genes in primary	13606 (59.01)	9452 (40.99)	
CRCR9P	EPHB1 in primary	375 (99.21)	3 (0.79)	ns	Mutated EPHB1	3 (0.79)	375 (99.21)	< 0.0001*
	All mutated genes in primary	15063 (99.62)	57 (0.38)		All mutated genes in primary	7899 (52.24)	7211 (47.76)	

Genes on panel (ctDNA): Detected genes on Cancer Panel by target sequencing. The cancer panel is consisted of common mutated genes betwen primary and recurrent sites.

*p-value indicated the statistical significance of mutated genes in ctDNA compared to primary specific mutations.

### Comparison of neoantigen presentation ability between commonly mutated genes and all mutated genes in primary tumors

We summarized the results of both factors to determine the ability to present neoantigen peptides in five cases (Tables 3 and 4). As shown in Table 2, CRCR4P, CRCR7P, CRCR8P, and CRCR9P showed significantly higher expression of ctDNA-detected mutated genes compared with other mutated genes in the primary tumor. However, these four cases showed no binding affinity to HLA (Table 4); therefore, none of the mutated genes in the four cases were presented as neoantigens. Furthermore, highly mutated genes were observed in both the primary and recurrent sites and were expressed as transcripts; however, they could not bind to HLA. Consequently, they could not be presented as neoantigens that activate the tumor immune response.

**Table 3:** Comparison of RNA expression of tumor specific mutated transcripts between ctDNA detected mutated genes and all mutated genes in primary tumor.

	Number of estimated peptides		
	No-Expression	Expression	Fisher
CRCR1P			
RNA exp. of ctDNA detected mutated genes in primary (%)	3794 (57.75)	2776 (42.25)	< 0.0001
RNA exp. of all mutated genes in primary (%)	12082 (70.31)	5103 (29.69)	
CRCR4P			
RNA exp. of ctDNA detected mutated genes in primary (%)	754 (66.49)	380 (33.51)	< 0.0001
RNA exp. of all mutated genes in primary (%)	1884 (31.66)	3552 (65.34)	
CRCR7P			
RNA exp. of ctDNA detected mutated genes in primary (%)	7260(45.75)	8610 (54.25)	< 0.0001
RNA exp. of all mutated genes in primary (%)	13585(53.34)	11885(46.66)	
CRCR8P			
RNA exp. of ctDNA detected mutated genes in primary (%)	2680(50.64)	2612(49.36)	< 0.0001
RNA exp. of all mutated genes in primary (%)	10559(59.43)	7207(40.57)	
CRCR9P			
RNA exp. of ctDNA detected mutated genes in primary (%)	396 (14.77)	2286 (85.23)	< 0.0001
RNA exp. of all mutated genes in primary (%)	7506 (58.57)	5310 (41.43)

**Table 4:** Comparison of the estimated binding affinity to HLA (IC50) between ctDNA detected mutated genes and all mutated genes in primary tumor.

	Number of estimated peptides		
	Low Affinity: IC50 > 50	High Affinity: IC50 < 50	Fisher
CRCR1P			
Neoantigens of ctDNA detected mutated genes in primary (%)	6519 (99.22)	51 (0.78)	
Neoantigens of all mutated genes in primary (%)	16987 (98.85)	198 (1.15)	p = 0.0109
CRCR4P			
Neoantigens of ctDNA detected mutated genes in primary (%)	1131 (99.74)	3 (0.26)	ns
Neoantigens of all mutated genes in primary (%)	5411 (99.54)	25 (0.46)	
CRCR7P			
Neoantigens of ctDNA detected mutated genes in primary (%)	15810 (99.62)	60 (0.38)	ns
Neoantigens of all mutated genes in primary (%)	25352 (99.54)	118 (0.46)	
CRCR8P			
Neoantigens of ctDNA detected mutated genes in primary (%)	5262 (99.43)	30 (0.57)	ns
Neoantigens of all mutated genes in primary (%)	17646 (99.32)	120 (0.68)	
CRCR9P			
Neoantigens of ctDNA detected genes in primary (%)	2674 (99.70)	8 (0.30)	ns
Neoantigens of all mutated genes in primary (%)	12764 (99.67)	52 (0.33)	

## Discussion

We found that the commonly mutated genes between primary and recurrent tumors indicated the expression of these transcripts, although there is no binding affinity to HLA. Therefore, these mutated genes were not induced neoantigens in the activation of the tumor-immune system. We assumed that the frequently mutated genes in recurrent tumors were derived from immune-tolerated clones in primary tumors without neoantigen presentation. Our previous study supports this finding. We compared the expression of tumor immune response-related genes, such as *CD8*, *CD4*, *PD-1*, *LAG3*, *A2aR,* and *TIM-3,* between primary and metastatic sites using the same RNA seq data from the same sample set used in the current study^14^. We found abundant expression of an immune exhausted indicator, *TIM-3*, in metastatic sites compared with that in primary sites in an in-house study as well as The Cancer Genome Atlas data^14^. In our previous study, we found that postoperative recurrence requires immune tolerance in the cancer microenvironment of colorectal cancer.

Meanwhile, we conducted targeted sequencing of ctDNA using the cancer panel comprising 416 commonly mutated genes between primary and recurrent sites. As a result, several genes, such as OR10A in CRCR1P and PEX5 in CRCR7P, revealed immune-tolerated findings without presentation of the neoantigens due to the mutually exclusive findings in either the loss of expression of mutated genes or lack of binding affinity to HLA. Immune tolerance induced by the loss of neoantigen presentation may be essential for clones to form recurrences. As we described above, commonly mutated clones between primary and recurrent sites indicated immune-resistant owing to the diminished binding affinity of neoantigen to HLA. In addition, the expression of immune exhausted genes, such as *TIM-3* was more abundant in the recurrent than primary sites in our previous study^14^. Wang et al.^19^ reported that Tim-3 inhibited the MHC-I-restricted antigen presentation not in cancer cells but in macrophages in vitro and in vivo. Regarding the cause of the reduced binding affinity to HLA in CRC, the loss of MHC class I expression plays a pivotal role in presenting processed antigens to T lymphocytes, including tumor antigens in colorectal cancer cases^20^, and LOH of HLA class I genes and B2M mutations have also been reported to be an indicator of poor prognosis^21,22^. Therefore, we assumed that most mutated genes in primary and recurrence sites detected by ctDNA have derived from the immune-resistant clones with the loss of MHC class I expression.

The limited number of target genes in each cancer panel was a limitation of the current study. We could not compare the detectability of ctDNA among the three groups, such as primary and recurrence commonly mutated genes, primary site-specific mutated genes, and recurrent site-specific mutated genes, owing to the limited number of plasma samples. In addition, we did not examine the binding affinity of estimated neoantigens to MHC-class II HLAs. Further study is required to elucidate the complete significance of the mutation in the plasma ctDNA. In addition, the detectability of mutated ctDNA preoperatively was low. We usually implement the target re-sequencing analysis using cancer panels of Foundation one, Gardant 360, and others carrying canonical driver genes. However, in the current study, we established and applied the cancer panel carrying commonly mutated genes between primary and recurrence tumors to comprehend the involvement of the host immunity during the evolutional process from primary to recurrence sites. Therefore, we could not detect the mutated ctDNA in the preoperative plasma samples.

In conclusion, recurrence required immune tolerance derived from the loss of neoantigen presentation ability, which was caused either by reduced cancer-specific mutated gene expression or by low binding affinity to HLA in CRC cases. The estimated neoantigen peptide derived from commonly mutated genes between primary and recurrent tumors showed no binding affinity to HLA compared with all mutated genes at primary sites.

## Materials and methods

### Enrolled patients and plasma samples

We used WES and RNA sequencing on ten primary tumors and ten postoperative metastatic tumors (the first one of metastases in each case) from ten cases of CRC from our previous study^14^ and established a cancer panel in the current study (Fig. S3). Therefore, we collected and examined 47 plasma samples from six cases of CRC: CRCR1, CRCR4, CRCR5, CRCR7, CRCR8, and CRCR9 (Table 1).

### Ethics statement

The study design was approved by the institutional review boards and ethics committees of the hospitals to which the patients were admitted (the Kyushu University Hospital Institutional Review Board [protocol number 609-06] and Cancer Institute Hospital Institutional Review Board [protocol number 2010-1058]). This study was conducted in accordance with the principles of the Declaration of Helsinki. Written informed consent was obtained from all study participants.

### Sample collection and preparation

Genomic DNA and RNA were extracted from freshly frozen tumor samples and adjacent normal intestinal mucosa using an AllPrep DNA/RNA Mini Kit (Qiagen, Hilden, Germany), according to the manufacturer’s instructions.

### Establishment of the cancer panel

We focused on the fundamental dynamics of the ctDNA fraction during the clinical course of CRC. The genome sequences of ten primary tumors and ten metastatic tumors were extracted, and exome sequencing was conducted (Table 1). According to the manufacturer's instructions, DNA was captured using a SureSelect Human All Exon 50 Mb kit (Agilent Technologies, Santa Clara, CA, USA). Captured DNA was sequenced using a HiSeq 2500 (Illumina K.K., Tokyo, Japan) with the paired-end 75–100-bp read option.

The commonly mutated gene of MAF in the primary site and the metastatic site was selected in each case for carrying on the customized cancer panel. In terms of establishing a cancer panel, we used ten primary sites and ten metastatic sites in our previous study (Table 1). We applied 451 mutated genes for the bespoke cancer panel (Fig. S3) established from commonly mutated genes between ten primary and ten metastatic sites. However, because of the inadequate amount of blood samples, we did not conduct a target sequence of plasma samples of CRCR2, CRCR3, CRCR 6, and CRCR10.

### Next-generation sequencing library construction

Indexed Illumina next-generation sequencing (NGS) libraries were prepared from plasma DNA. Plasma DNA was used for library construction without additional fragmentation. Genomic DNA was sheared before library construction using a Covaris S2 instrument (Woburn, MA, USA) to obtain 200-bp fragments. According to the manufacturer's protocol, NGS libraries of plasma DNA were constructed using the KAPA Hyper Prep Kit (Kapa Biosystems, Wilmington, MA, USA). A sequencing library was prepared using the KAPA Hyper Prep Kit (Kapa Biosystems) and SureSelect Target Enrichment System (Agilent Technologies). End repair and A-tailing reactions were performed in 60-µL reaction volumes. The mixtures were then incubated at 20 °C and 65 °C for 30 min each. Adapter ligation was performed using 110-µL volumes, and samples were incubated at 16 °C for 16 h using a SureSelect Adapter (Agilent Technologies). After postligation cleanup, the ligated fragments were amplified in a 50-µL solution containing 2 × KAPA HiFi HotStart ReadyMix and 10 × KAPA Library Amplification Primer Mix (Kapa Biosystems). We used the following cycling protocol: 98 °C for 45 s, 14–16 cycles (depending on the input DNA mass) of 98 °C for 15 s, 65 °C for 30 s, 72 °C for 30 s, and 72 °C for 5 min (1 cycle). Library purity, library concentration, and fragment length were determined using a 2100 Bioanalyzer (Agilent Technologies).

### Targeted sequencing

Plasma DNA extracted from CRC patient samples was captured using a SureSelectXT Custom 1 Kb–499 kb, 16 (Agilent Technology) according to the manufacturer’s instructions. A panel of 451 genes was designed and validated in this study. Captured DNA was sequenced using a HiSeq2000 (Illumina K.K.) to generate paired-end (75–100 bp) reads for each sample. Targeted deep sequencing was performed for all samples using a multigene panel, with a mean sequencing depth of 3810×.

### Mutation calling

We used WES data from our previous study^14^. The sequence data were processed using an in-house pipeline (https://genomon-project.github.io/GenomonPagesR/). The sequencing reads were aligned to the National Center for Biotechnology Information Human Reference Genome Build 37 hg19 with BWA version 0.7.8 using the default parameters. Polymerase chain reaction duplicates were removed using the Picard method. Mutation calling was performed using the EBCall algorithm^23^ with the following parameters: (1) mapping quality score ≥ 20; (2) base quality score ≥ 15; (3) both the tumor and normal depths ≥ 10; (4) variant reads in tumors ≥ 4; (5) variant allele frequencies (VAFs) in tumor samples ≥ 0.02; and (6) VAFs in normal samples ≤ 0.01.

### RNA sequencing

We used RNA sequencing data from our previous study^14^; however, we applied RNA seq data from six primary sites and six metastatic sites (black boxes in Table 1). Approximately three billion single-end reads were generated using an Illumina HiSeq 2500 system, as previously described^24^.

### Data availability statement

Data are available at: https://humandbs.biosciencedbc.jp/en/hum0120-v4#target2. Our sequence data are available as NBDC Research ID; hum0120.v4. In terms of mutated ctDNA, we can obtain target sequence data of ctDNA (JGAS000549). In addition, whole exome sequences of 10 primary sites and metastatic sites (9 liver tumors and 5 lung tumors) were available at: Tumor tissues (DRA011183) and non-tumor tissue non-tumor tissues (JGAD000311).

### HLA genotyping (Hayashi method)

For HLA genotyping from whole-genome sequencing data, the Bayesian ALPHLARD method was used, which was designed to perform accurate HLA genotyping from short-read data and predict the HLA sequences of the sample. The latter function enables the identification of somatic mutations by comparing the HLA sequences of the tumor and matched normal samples. The statistical formulation for the posterior probability can be described as follows:

$$ {\text{P }}\left( {{\text{R, S, I}}|{\text{ X}}} \right)\, \propto \,{\text{P }}\left( {{\text{X }}|{\text{ S, I}}} \right){\text{ P }}\left( {\text{I}} \right){\text{ P}}({\text{R, S}}) $$

where R = (R1, R2) is the pair of HLA types (reference sequences), *S* = (*S*1, *S*2) is the pair of sample HLA sequences, *X* = (× 1, × 2,…) is a set of sequence reads, and *I* = (*I*1, *I*2,…) is a set of variables using one or two values (jth element; I_j_, indicating that the jth read x_j_ is generated from *S*_*I j*_). On the right-hand side of the equation, the left term indicates the likelihood of the sequence reads when the HLA and reference sequences are fixed. The middle and right times are the priors. The parameters, HLA sequences, and HLA types were determined using the Markov Chain Monte Carlo procedure.

### Prediction of potential N-acetylglucosamine peptides

Using the Neoantimon package in R, the HLA types of individual patients were obtained (Fig. S3). To identify potential N-acetylglucosamine (NAG) peptides, we used a nonrelapse-based automated pipeline, available at https://github.com/hase62/Neoantimon. Using WES data, this pipeline can easily and automatically construct mutated, and wild-type peptides, including the mutation position, calculation of binding affinity to MHC molecules (using netMHCpan4.0), and integration of the total and tumor-specific RNA expression data based on VAFs calculated from RNA sequence data at the mutation position.

#### Institutional review board statement

The study design was approved by the institutional review boards and ethics committees of the hospitals to which the patients were admitted (the Kyushu University Hospital Institutional Review Board [protocol number 609-06] and Cancer Institute Hospital Institutional Review Board [protocol number 2010-1058]). This study was conducted in accordance with the principles of the Declaration of Helsinki.

#### Informed consent statement

Written informed consent was obtained from all study participants.

### Statistical analyses

We used the Mann–Whitney U test or Fisher’s exact tests to test the associations between variables. Data analyses were performed using JMP 14 (SAS Institute, Cary, NC, USA) and R software version 3·1·1 (R Foundation for Statistical Computing, Vienna, Austria).

## Supplementary Information

Supplementary Figure S1.

Supplementary Figure S2.

Supplementary Figure S3.
