# Supplementary material for: Mutated genes on ctDNA detecting postoperative recurrence presented reduced neoantigens in primary tumors in colorectal cancer cases
Source: Sci Rep. 2023 Jan 24;13:1366. doi: 10.1038/s41598-023-28575-3 (PMC9873919; doi:10.1038/s41598-023-28575-3)
Supplement: Supplementary file 1 — Supplementary Figure S1. [file 41598_2023_28575_MOESM1_ESM.pdf]

Fig. S1

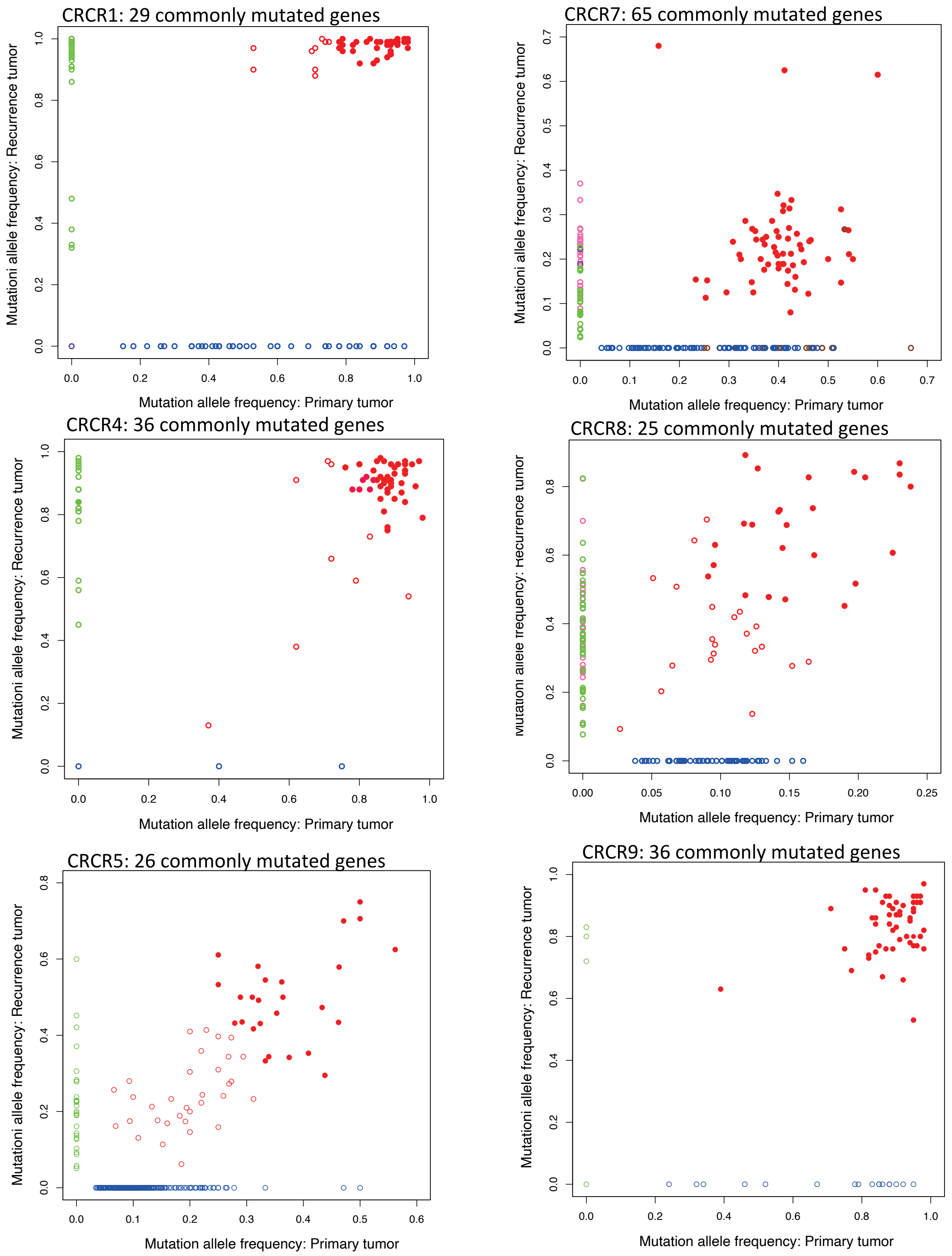

Fig. S1: Selected mutated genes for target sequence of liquid biopsy on the customized cancer panel.

The horizontal and vertical axes indicated the allele frequency of mutated genes in the primary and metastatic sites, respectively. Solid red spots indicate the selected commonly frequent mutated genes on the customized cancer panel.
