# Supplementary material for: Mutated genes on ctDNA detecting postoperative recurrence presented reduced neoantigens in primary tumors in colorectal cancer cases
Source: Sci Rep. 2023 Jan 24;13:1366. doi: 10.1038/s41598-023-28575-3 (PMC9873919; doi:10.1038/s41598-023-28575-3)
Supplement: Supplementary file 2 — Supplementary Figure S2. [file 41598_2023_28575_MOESM2_ESM.pdf]

Fig. S2

Number of mutated genes on the cancer panel

CRCR1	29 genes
CRCR2	28 genes
CRCR3	54 genes
CRCR4	36 genes
CRCR5	26 genes
CRCR6	24genes
CRCR7	65 genes
CRCR8	25 genes
CRCR9	36 genes
CRCR10	120 genes
Total	**443 genes**

+

TCGA significant mutated genes

APC	FAM123B	CDC27	ELF3	PCDHGA9
TP53	PIK3CA	KIAA1804	CRTC1	RBM10
KRAS	TCF7L2	PCDHGB1	CASP8	PTEN
NRAS	ARID1A	PCBP1	CACNG3	DNMT1
SMAD4	BRAF	ZHX2	PCDHGA7	BCOR
FBXW7	MGC42105	PCDHA3	CCBP2	PCDHGB5
SMAD2	ACVR1B	PCDHA2	MAP2K4	ZFP3

35 genes

451 mutated genes

Fig. S2: The number of genes on the customized cancer panel.
We selected 443 commonly mutated genes between 10 primary sites and ten recurrent (metastatic) sites to establish a cancer panel for target resequencing. In addition, we added 35 established significant mutated genes from TCGA to the bottom of the customized cancer panel to avoid missed ctDNA detection. Eventually, we selected 451 genes for the bespoke cancer panel.
