# Supplementary material for: Mutated genes on ctDNA detecting postoperative recurrence presented reduced neoantigens in primary tumors in colorectal cancer cases
Source: Sci Rep. 2023 Jan 24;13:1366. doi: 10.1038/s41598-023-28575-3 (PMC9873919; doi:10.1038/s41598-023-28575-3)
Supplement: Supplementary file 3 — Supplementary Figure S3. [file 41598_2023_28575_MOESM3_ESM.pdf]

Fig. S3

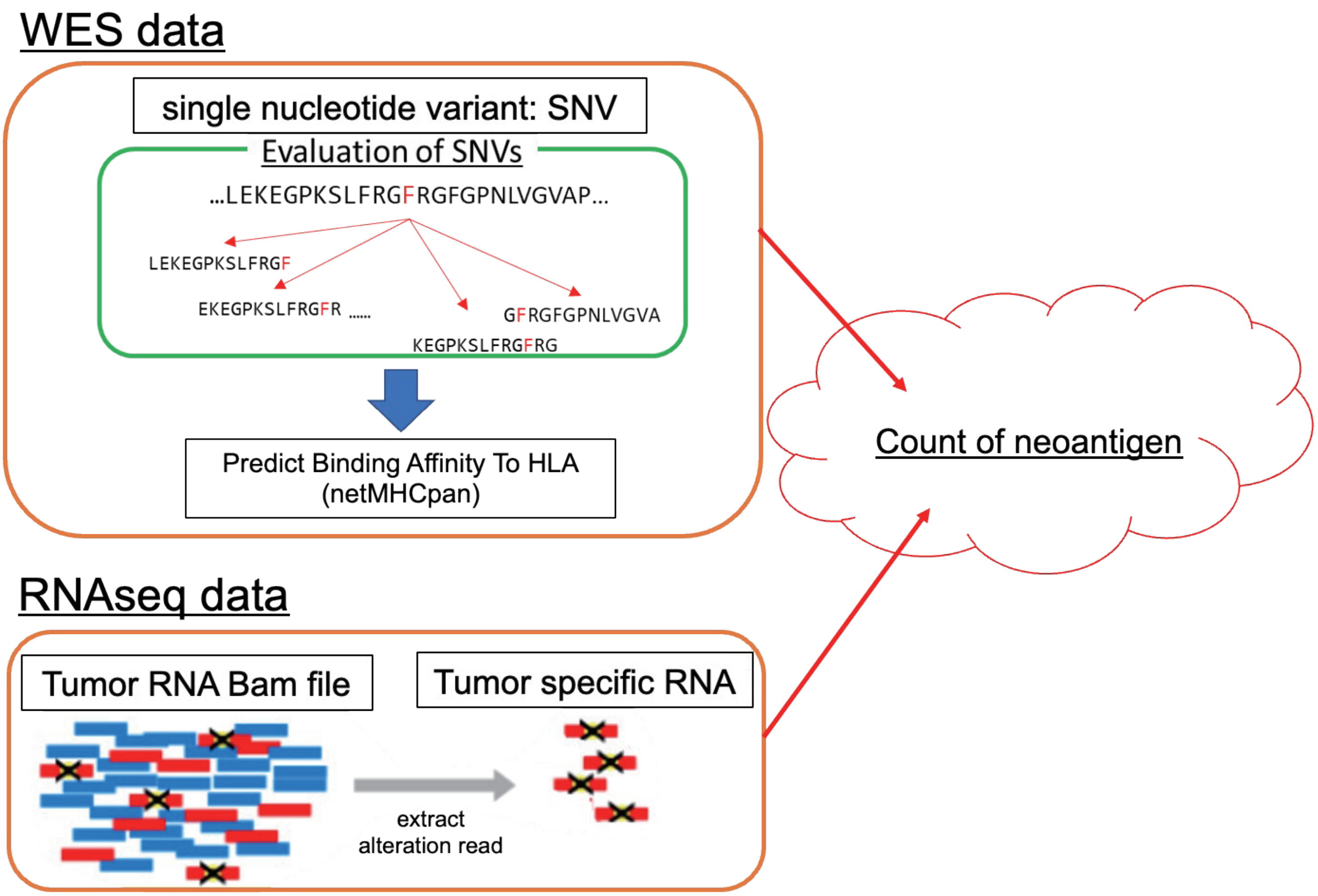

Fig. S3: Neoantigen analysis of mutated ctDNA.

Initially, we evaluated mutations to estimate neoantigen peptides in detected ctDNA at 47 points using the Neoantimon R package. Then, we estimated the binding affinity of every peptide bound to HLA, which consisted of six haplotypes (HLA-A, -B, -C) according to netMHCpan. The pipelines were established and calculated. Meanwhile, we calculated "tumor-specific RNA" using extracted altered readouts of the whole primary tumor RNA BAM file.
